# Rate of Entropy Production in Stochastic Mechanical Systems

**DOI:** 10.3390/e24010019

**Published:** 2021-12-23

**Authors:** Gregory S. Chirikjian

**Affiliations:** Department of Mechanical Engineering, National University of Singapore, Singapore 117575, Singapore; mpegre@nus.edu.sg

**Keywords:** entropy, information, inequalities, Lie Group, statistical mechanics, fluctuation-dissipation theory, covariance, stochastic modeling

## Abstract

Entropy production in stochastic mechanical systems is examined here with strict bounds on its rate. Stochastic mechanical systems include pure diffusions in Euclidean space or on Lie groups, as well as systems evolving on phase space for which the fluctuation-dissipation theorem applies, i.e., return-to-equilibrium processes. Two separate ways for ensembles of such mechanical systems forced by noise to reach equilibrium are examined here. First, a restorative potential and damping can be applied, leading to a classical return-to-equilibrium process wherein energy taken out by damping can balance the energy going in from the noise. Second, the process evolves on a compact configuration space (such as random walks on spheres, torsion angles in chain molecules, and rotational Brownian motion) lead to long-time solutions that are constant over the configuration space, regardless of whether or not damping and random forcing balance. This is a kind of potential-free equilibrium distribution resulting from topological constraints. Inertial and noninertial (kinematic) systems are considered. These systems can consist of unconstrained particles or more complex systems with constraints, such as rigid-bodies or linkages. These more complicated systems evolve on Lie groups and model phenomena such as rotational Brownian motion and nonholonomic robotic systems. In all cases, it is shown that the rate of entropy production is closely related to the appropriate concept of Fisher information matrix of the probability density defined by the Fokker–Planck equation. Classical results from information theory are then repurposed to provide computable bounds on the rate of entropy production in stochastic mechanical systems.

## 1. Introduction

The second law of thermodynamics introduced the concept of entropy, and states that at the macroscopic scale entropy is nondecreasing for a closed isolated system, i.e., one for which no heat enters or leaves and on which no external work is performed. This law was postulated in the mid 1800s, motivated in part by the desire for efficiency gains in steam engines. A closed system could be an isolated container on a lab bench, or the whole universe, leading to the famous phrase “the universe tends toward disorder” which is a paraphrase of a statement made by Rudolf Clausius circa 1865.

Statistical mechanical arguments were developed to explain the second law at the microscopic/molecular level and were established in large part by Ludwig Boltzmann’s classical work for ideal gases involving deterministic collision models between particles. In their famous H-theorem, Boltzmann proved that:

$$\frac{dH}{dt}\;\leq 0$$

where

$$H\left(t\right)\;≐\;\int_{R^{3}}f\left(v,t\right)\log f\left(v,t\right)\;dv$$

with f(v,t) being the velocity distribution of gas particles in a container at a particular value of time, *t*, under the assumption of isotropic spatial distribution. This expression is related to the more general Gibbs formula for entropy given later in this introduction, which differs in sign and scale. As *t* increases, f(v,t) converges to an equilibrium distribution, $f_{\infty }\left(v\right)$, known as the Maxwell–Boltzmann distribution, where *H* is minimized (or entropy is maximized) for a given value of temperature.

A completely different approach to Boltzmann’s is the *stochastic mechanical system* in which each element in the statistical ensemble is a classical mechanical system which is forced by a combination of conservative forces, random Brownian motion and viscous damping. A stochastic mechanical system is modeled using stochastic differential equations, and ensemble behavior is described by the corresponding Fokker–Planck equation. They are not limited to particle systems, and can either be modelled as having inertia or can be purely kinematic. They can be complex molecules or even systems with nonholonomic kinematic constraints such as robots.

The main contributions of this paper are as follows:

(1) The recognition that stochastic mechanical models can be used in place of the original deterministic collision models used to formulate statistical mechanics;
(2) The interpretation of these stochastic models as Itô or Stratonovich is irrelevant for systems that have nonzero mass, but that these interpretations provide different results as mass becomes zero—an effect almost never discussed in statistical mechanical works;
(3) Conditions for a stochastic mechanical system with configuration-dependent noise and damping to reach equilibrium (i.e., the time-independent probability distribution on phase space) are established, generalizing the Einstein relations and providing a statement that is new and different than those for detailed balance, and represents a new observation in fluctuation-dissipation theory;
(4) The aforementioned stationary pdf to which a stochastic mechanical ensemble converges is in fact the Boltzmann distribution from statistical mechanics, which is obtained via a different path than it was in statistical mechanics;
(5) Novel solutions to diffusion equations on Lie groups are provided;
(6) Inequalities from information theory are extended beyond their original setting (e.g., to include diffusion processes on Lie groups in addition to Euclidean spaces) and used to bound the entropy and rate of entropy production in stochastic mechanical systems such as the rigid Brownian rotor and mobile robots, which are beyond the scope of classical statistical mechanics.

Mixed in with these new results is a substantial amount of review material, since it makes little sense to present intricacies about differences between Itô and Stratonovich stochastic differential equations or to talk about diffusions on Lie groups to someone familiar only with statistical mechanics or information theory. Moreover, despite the similarities in the form of entropy in these fields, the inequalities of information theory are rarely known in statistical mechanics, and the concept of Hamiltonian mechanics and phase space which are essential in statistical mechanics may be unknown to those familiar with information theory.

Stochastic mechanical models (i.e., stochastic differential equations and associated Fokker–Planck equations) are examined here with the goal of establishing strict bounds on the rate of entropy production. Individual stochastic trajectories can be generated numerically from a stochastic differential equation, but it is an ensemble of such trajectories that conveys important statistical information about the system. These ensembles can be used to generate a family of histograms indexed by time, or the associated Fokker–Planck equation can be solved to obtain the same family of probability densities. Regardless of which approach is taken, each stochastic mechanical systems has an associated family of probability density functions (indexed by time) from which entropy and its rate can be computed.

A probability density function (pdf) on a measure space (X,μ) which is indexed by time is a function $f:X\times R_{\geq 0}\;⟶\;R_{\geq 0}$ such that:

$$\int_{X}f\left(x,t\right)\;d\mu \left(x\right)\;=\;1\;.$$

The entropy of *f* at each value of time is defined as:

(1) $$S\left(t\right)\;≐\;-\int_{X}f\left(x,t\right)\log f\left(x,t\right)\;d\mu \left(x\right)\;.$$

The choice of base of the logarithm amounts to a choice of measurement units for *S*, as a different base will result in a different scaling of S(t). Throughout this article, $\log≐\log_{e}=\ln$.

When considering a statistical mechanical system, the entropy of a time-varying probability density on phase space is defined using Gibb’s formula as:

(2) $$S_{B}\left(t\right)\;≐\;-k_{B}\int_{q}\int_{p}f\left(p,q,t\right)\log f\left(p,q,t\right)\;dp\;dq$$

where $q\in Q\subseteq R^{n}$ is a set of coordinates, and $p\in R^{n}$ are the corresponding conjugate momenta. The Lebesgue measure on the 2n-dimensional phase space, $dp\;dq=dq_{1}...dq_{n}dp_{1}...dp_{n}$, is invariant under coordinate changes, though such changes do affect the bounds of integration *Q*, unless $Q=R^{n}$. The inclusion of the Boltzmann constant $k_{B}$ in (2) is for consistency with the statistical mechanics literature.

It should be noted that this integral is an approximation of a sum over microstates, the size of which is specified by the Planck scale. Whereas mathematically speaking continuous entropy has no lower bound, for physical systems it does because discrete entropy computed by summing over microstates is always nonnegative. As long as the features of the probability density function (pdf) are coarser than the Planck scale, then entropy differences using this continuous integral formula and the analogous entropy formula computed by summing over discrete states will be the same. That is, $S_{cont}\;\neq \;S_{disc}$, but $\Delta S_{cont}\;=\;\Delta S_{disc}$ for physical systems.

In information theory, entropy can also be over a continuous space (in which case it is called “differential” entropy) or it can be defined over a discrete space, such as a set of symbols. It is no coincidence that the same expression (without the factor of $k_{B}$) appears with the same name in information theory. As the folklore goes, the founder of information theory, Claude Shannon had not yet fixed the name ‘entropy’, and had a conversation with mathematician John von Neumann, who suggested [1,2]:

“You should call it entropy, for two reasons. First, your expression has been used in statistical mechanics under that name. Second, nobody really knows what entropy is, so in a debate you will always have the advantage.”

These words are not exact because it was supposedly a phone conversation (if it ever happened at all) subsequently relayed through other people.

The configurational probability density is the marginal distribution:

(3) $$f\left(q,t\right)\;≐\;\frac{1}{\sqrt{M(q)}}\int_{p}f\left(p,q,t\right)\;dp$$

which is defined in this way so that:

$$\int_{q\in Q}f\left(q,t\right)\;\sqrt{M(q)}\;dq\;=\;1\;.$$

The corresponding configurational entropy is:

(4) $$S_{Q}\left(t\right)\;≐\;-\int_{q\in Q}f\left(q,t\right)\log f\left(q,t\right)\;\sqrt{M(q)}\;dq$$

where M(q) is the metric tensor for the configuration space, which is the mass matrix in the case of a mechanical system with inertia.

A unimodular Lie group is one with an integration measure that is invariant under shifts from the left and the right. In the case when q parameterizes a unimodular Lie group, *G*, such as the group of rotations or the Euclidean motion group, then the entropy of a time-indexed pdf $f:G\times R_{\geq 0}\;\to \;R_{\geq 0}$ is:

(5) $$S_{G}\left(t\right)\;≐\;-\int_{G}f\left(g,t\right)\log f\left(g,t\right)\;dg$$

where the Haar measure dg takes on different appearances under changes of parametrization but the value of the integral is independent of parametrization and it is invariant under shifts of the form $g\to g_{0}g$ and $g\to gg_{0}$ for any fixed $g_{0}\in G$.

The main topic of this article is to study the rate of entropy increase in stochastically forced mechanical systems. That is, for any of the entropies defined above, to calculate or bound $\dot{S}$. In particular, by simply moving the time derivative inside the integral and using the product rule from Calculus,

(6) $$\dot{S}\left(t\right)\;=\;-\int_{X}\left\{\frac{\partial f}{\partial t}\log f+\frac{\partial f}{\partial t}\right\}\;d\mu \left(x\right).$$

However, since f(x,t) is a probability density function at each value of time, whose integral is equal to 1,

$$\int_{X}\frac{\partial f}{\partial t}\;d\mu \left(x\right)=\frac{d}{dt}\int_{X}f\left(x,t\right)\;d\mu \left(x\right)=0,$$

and so the second term in the above braces integrates to zero. Consequently:

(7) $$\dot{S}\left(t\right)\;=\;-\int_{X}\frac{\partial f}{\partial t}\log f\;d\mu \left(x\right).$$

It is also possible to bound the value of entropy itself. The function Φ(x)=−logx is a convex function. Consequently, Jensen’s inequality gives:

$$S\;=\;\int_{X}f\left(x\right)\Phi \left(f\left(x\right)\right)d\mu \left(x\right)\;\geq \;\Phi \left({∥f∥}^{2}\right)$$

or

(8) $$S\;\geq \;-\log\left({∥f∥}^{2}\right)$$

where

$${∥f∥}^{2}\;≐\;\int_{X}{\left|f\left(x\right)\right|}^{2}d\mu \left(x\right)\;.$$

As a consequence of Parseval’s inequality, (8) can then be stated in Fourier space. This is true not only for the case of Euclidean space, but for wide classes of unimodular Lie groups [3].

Moreover, S(t) can be bounded from above in some contexts. For example, on Euclidean spaces it is well known that Gaussian distributions have maximum entropy over all pdfs with a given mean and covariance. Therefore, if f(x,t) is an arbitrary time-evolving pdf on $R^{d}$ with mean $\mu_{f}\left(t\right)$ and covariance $\Sigma_{f}\left(t\right)$, and if $\rho_{\mu ,\Sigma }\left(x\right)$ denotes a Gaussian distribution with mean μ and covariance Σ, then:

(9) $$S_{f}\left(t\right)\;\leq \;S_{\rho_{\mu_{f},\Sigma_{f}}}\left(t\right)\;.$$

On a compact space such as the circle or rotation group, the uniform distribution has the absolute maximum entropy of all distributions on those spaces.

## 2. Rate of Entropy Production for Stochastic Processes on Euclidean Space

The stochastic mechanical models addressed here are described as stochastic differential equations forced by Brownian noise (i.e., Gaussian white noise, or equivalently, increments of Wiener processes).

### 2.1. Review of Stochastic Differential Equations

Stochastic differential equations (SDEs) are forced by Gaussian white noises $dw_{i}$, which are increments of uncorrelated unit-strength Weiner processes. These define Brownian motion processes. That is, each $dw_{i}\left(t\right)$ can be viewed as an independent random draw from a one-dimensional Gaussian distribution with zero mean and unit variance. Let *d* denote the dimension of a Euclidean space on which the random process evolves. For systems with inertia, d=2n is phase space, and for noninertial systems d=n. The independent uncorrelated unit strength white noises $dw_{i}$ form the components of a vector $dw\in R^{d}$. For example, given the stochastic differential equation on $R^{d}$

dx=Bdw

where $B\in R^{d\times d}$ is a constant full-rank matrix, the distribution f(x,t) describing the ensemble of an infinite number of trajectories will satisfy:

$$\frac{\partial f}{\partial t}\;=\;\frac{1}{2}\sum_{i,j=1}^{d}D_{ij}\frac{\partial^{2}f}{\partial x_{i}\partial x_{j}}\;.$$

where $D=\left[D_{ij}\right]=BB^{T}$. The solution of this equation subject to initial conditions f(x,0)=δ(x−0) is the time-varying Gaussian distribution:

(10) $$f\left(x,t\right)\;=\;\frac{1}{{\left(2\pi \right)}^{d/2}{\left|Dt\right|}^{1/2}}\exp\left(-\frac{1}{2}x^{T}{\left(Dt\right)}^{-1}x\right)$$

where |A| denotes the determinant of a square matrix *A*. The entropy for this can be computed in closed form as: [4]

(11) $$S\left(t\right)\;=\;\log\left\{{\left(2\pi e\right)}^{d/2}{\left|\Sigma \left(t\right)\right|}^{1/2}\right\}\;.$$

where Σ(t)=Dt in this context. More generally (11) can be used as the upper bound in (9) with $\Sigma =\Sigma_{f}$ since entropy is independent of the mean.

As an example of (8), applying it to a Gaussian distribution on $R^{d}$ gives:

$$S\left(t\right)\;\geq \;\log\left\{{\left(4\pi \right)}^{d/2}{\left|Dt\right|}^{1/2}\right\}.$$

Comparing with the exact expression in (11) verifies this since 4π<2πe and log(x) is a monotonically increasing function.

There are two major kinds of stochastic differential equations (SDEs), Itô and Stratonovich. Both are forced by Gaussian white noises $dw_{i}$. In the simple example above, Itô and Stratonovich interpretations lead to the same result, but in more complex cases where the coupling matrix *B* is configuration-dependent, the two intepretations will differ. A brief review of the main features of these two different interpretations of SDEs is given here based on the longer exposition in [5,6].

Historically, the Itô interpretation came first. If

(12) $$dx_{i}\left(t\right)=a_{i}\left(x_{1}\left(t\right),...,x_{d}\left(t\right),t\right)dt+\sum_{j=1}^{m}B_{ij}\left(x_{1}\left(t\right),...,x_{d}\left(t\right),t\right)\;dw_{j}\left(t\right)\;\mathrm{for}\;i=1,...,d$$

is an Itô SDE describing a random process on $R^{d}$, where now $B\in R^{d\times m}$, then the corresponding Fokker–Planck equation governing the probability density of the ensemble of states, f(x,t), is [5,6]

(13) $$\frac{\partial f(x,t)}{\partial t}=-\sum_{i=1}^{d}\frac{\partial }{\partial x_{i}}\left(a_{i}\left(x,t\right)f\left(x,t\right)\right)+\frac{1}{2}\sum_{i,j=1}^{d}\frac{\partial^{2}}{\partial x_{i}\partial x_{j}}\left(\sum_{k=1}^{m}B_{ik}\left(x,t\right)B_{kj}^{T}\left(x,t\right)f\left(x,t\right)\right),$$

Itô SDEs are popular in mathematical statistics contexts, measurement and filtering theory, and in finance, because of the ease with which expectations can be taken, and the associated martingale properties. In engineering contexts when modeling physical processes the Stratonovich interpretation of SDEs described below is more popular because standard rules of Calculus can be used [7,8]. For this reason the Stratonovich interpretation is popular in differential geometric contexts since moving between coordinate patches involves Calculus operations. A Stratonovich SDE describing the exact same random process as the Itô SDE given above is written as

(14) $$dx_{i}\left(t\right)=a_{i}^{s}\left(x_{1}\left(t\right),...,x_{d}\left(t\right),t\right)dt+\sum_{j=1}^{m}B_{ij}\left(x_{1}\left(t\right),...,x_{d}\left(t\right),t\right)\;Ⓢ\;dw_{j}\left(t\right)\;\mathrm{for}\;i=1,...,d$$

where Ⓢ is used to denote the Stratonovich interpretion of the SDE, which differs from the Itô version. These interpretations are interchangeable with drift terems related as:

(15) $$a_{i}\left(x,t\right)=a_{i}^{s}\left(x,t\right)+\frac{1}{2}\sum_{j=1}^{m}\sum_{k=1}^{d}\frac{\partial B_{ij}}{\partial x_{k}}B_{kj}\;.$$

This illustrates that there is very simple way to interconvert between Itô and Stratonovich by either adding or subtracting the last term in (15).

The Stratonovich form of the Fokker–Planck Equation (FPE) is written as [5,6]:

(16) $$\frac{\partial f}{\partial t}=-\sum_{i=1}^{d}\frac{\partial }{\partial x_{i}}\left(a_{i}^{s}f\right)+\frac{1}{2}\sum_{i,j=1}^{d}\frac{\partial }{\partial x_{i}}\left[\sum_{k=1}^{m}B_{ik}\frac{\partial }{\partial x_{j}}\left(B_{jk}f\right)\right]$$

When *B* is independent of the configuration variable x, Itô snd Stratonovich versions of the FPE are always the same, as can be seen from (15) where the discrepancy between the two versions of drift term vanishes as the partial derivatives of *B* with respect to x vanish. This is a sufficient condition for Itô and Stratonovich SDEs to yield the same FPE, but is not a necessary for this to be the case, as will be seen in later examples. Similar equations hold for processes evolving on manifolds and Lie groups, as will be discussed later in the paper.

The main topic addressed here is the rate at which S(t) changes. This can be observed by substituting the solution of the Fokker–Planck equations into the definition of $\dot{S}$ in (6).

### 2.2. Rate of Entropy Production

Using the Stratonovich form of the FPE in (16), we arrive at the following theorem:

**Theorem** **1.** *The rate of entropy production for f(x,t) governed by (16) evolving freely on Euclidean space is positive and bounded from below by*

(17) $$\dot{S}\;\geq \;\frac{1}{2}\int_{R^{d}}\mathrm{trace}\left[\frac{\left(B{\left(x,t\right)}^{T}\nabla f\right){\left(B{\left(x,t\right)}^{T}\nabla f\right)}^{T}}{f}\right]\;dx$$

*when*

(18) $$\frac{\partial }{\partial x_{i}}\left(a_{i}^{s}-\sum_{k,j}B_{ik}\frac{\partial B_{jk}}{\partial x_{j}}\right)\geq 0$$

*with equality holding when*

(19) $$a_{i}^{s}-\sum_{k,j}B_{ik}\frac{\partial B_{jk}}{\partial x_{j}}=c_{i}$$

*is constant for all values of i. In the case when B is a constant and $D≐BB^{T}$, then*

(20) $$\dot{S}\;=\;\frac{1}{2}\mathrm{trace}\left[DF(t)\right]\;.$$

*where*

$$F\left(t\right)=\int_{R^{d}}\frac{\left(\nabla f\right){\left(\nabla f\right)}^{T}}{f}\;dx$$

*is the Fisher information matrix of f(x,t).*

**Proof.**  The ∂f/∂t term in

$$\dot{S}\;=\;-\int_{R^{d}}\frac{\partial f}{\partial t}\log f\;dx$$

can be expressed in terms of spatial derivatives by substituting in the FPE. This is written as two terms using integration by parts with the surface terms at infinity vanishing due to the fact that the process evolves freely and the pdf must decay to zero at infinity. First,

$$-\int_{R^{d}}\left\{-\sum_{i=1}^{d}\frac{\partial }{\partial x_{i}}\left(a_{i}^{s}f\right)\right\}\log f\;dx\;=\;-\int_{R^{d}}\sum_{i=1}^{d}a_{i}^{s}\frac{\partial f}{\partial x_{i}}\;dx\;.$$

Second,

$$-\int_{R^{d}}\left\{\frac{1}{2}\sum_{i,j=1}^{d}\frac{\partial }{\partial x_{i}}\left[\sum_{k=1}^{m}B_{ik}\frac{\partial }{\partial x_{j}}\left(B_{jk}f\right)\right]\right\}\log f\;dx\;=\;\frac{1}{2}\int_{R^{d}}\frac{1}{f}\sum_{i,j=1}^{d}\left[\sum_{k=1}^{m}B_{ik}\frac{\partial }{\partial x_{j}}\left(B_{jk}f\right)\right]\frac{\partial f}{\partial x_{i}}\;dx\;.$$

Expanding

$$\frac{\partial }{\partial x_{j}}\left(B_{jk}f\right)=\frac{\partial B_{jk}}{\partial x_{j}}f+B_{jk}\frac{\partial f}{\partial x_{j}}$$

and recollecting terms gives

$$\dot{S}\;=\;\int_{R^{d}}\sum_{i=1}^{d}\left\{-a_{i}^{s}+\sum_{i,j=1}^{d}B_{ik}\frac{\partial B_{jk}}{\partial x_{j}}\right\}\frac{\partial f}{\partial x_{i}}dx\;+\;\frac{1}{2}\int_{R^{d}}\frac{1}{f}\sum_{k=1}^{m}\left(\sum_{i=1}^{d}B_{ik}\frac{\partial f}{\partial x_{i}}\right)\left(\sum_{j=1}^{d}B_{jk}\frac{\partial f}{\partial x_{j}}\right)dx\;.$$

The second integral is always nonnegative, as it is a positive semi-definite quadratic form and can be written as:

$$\frac{1}{2}\mathrm{trace}\left[\int_{R^{d}}\frac{\left(B{\left(x,t\right)}^{T}\nabla f\right){\left(B{\left(x,t\right)}^{T}\nabla f\right)}^{T}}{f}dx\right]\;.$$

The first term can have either sign. However, if

$$a_{i}^{s}-\sum_{k,j}B_{ik}\frac{\partial B_{jk}}{\partial x_{j}}=c_{i}$$

is constant, then the first integral will vanish, and if by integration by parts the derivative in the first term is transferred over, the condition in the statement of the theorem will result since f≥0. □

When (18) holds it is clear that a looser lower bound than (17) akin to (20) can be obtained as:

(21) $$\dot{S}\;\geq \;\frac{1}{2}\mathrm{trace}\left[D_{0}\left(t\right)F\left(t\right)\right]$$

by constructing positive definite matrix $D_{0}\left(t\right)=D_{0}^{T}\left(t\right)$ such that the following matrix inequality:

$$D_{0}\left(t\right)\;\leq \;B\left(x,t\right)B^{T}\left(x,t\right)\;\;\;\forall \;\;x\in R^{d}$$

is satisfied. The reason why (21) then holds is because the trace is linear and the trace of the product of positive semi-definite matrices is nonnegative, and both *F* and $BB^{T}-D_{0}$ are positive semi-definite.

The condition (19) and result (20) are for diffusion processes with constant diffusion tensor and drift. Given initial conditions $f\left(x,0\right)=f_{0}\left(x\right)$, the solution f(x,t) in this case will be of the form:

(22) $$f\left(x,t\right)\;=\;\left(f_{0}*\rho_{ta,tBB^{T}}\right)\left(x\right)$$

where $\rho_{\mu \;,\Sigma }\left(x\right)$ is a multivariate Gaussian with mean μ and covariance Σ. The convolution of any two functions $f_{1},f_{2}\in \left(L^{1}\cap L^{2}\right)\left(R^{d}\right)$ is defined as:

(23) $$\left(f_{1}*f_{2}\right)\left(x\right)\;≐\;\int_{R^{d}}f_{1}\left(y\right)f_{2}\left(x-y\right)\;dy\;.$$

Fisher information plays an important part in probability theory [9,10] and its connections to physics also have been recognized [11,12,13]. For a recent review of its properties see [14].

Several inequalities from Information Theory can then be used to bound both entropy and entropy rate by quantities that are easily computable. For example, it is known that [15,16,17]:

(24) $$\frac{1}{\mathrm{tr}[F\left(f_{1}*f_{2}\right)P]}\;\geq \;\frac{1}{\mathrm{tr}[F\left(f_{1}\right)P]}+\frac{1}{\mathrm{tr}[F\left(f_{2}\right)P]}$$

where *P* is any real positive definite symmetric matrix with the same dimensions as *F*. When P=D this can then be used to give a lower bound on tr[FD], and hence on $\dot{S}$. Moreover, one reason for the significance of (24) in information theory is that it provides a path for proving the entropy power inequality [15,16] described below.

The entropy power of a pdf f(x) on $R^{d}$ was defined by Shannon as [4,18]:

$$N\left(f\right)\;≐\;\frac{\exp(2S(f)/d)}{2\pi e}$$

where S(f) denotes the entropy of *f*. The entropy power inequality is:

(25) $$N\left(f_{1}*f_{2}\right)\;\geq \;N\left(f_{1}\right)+N\left(f_{2}\right)\;.$$

Since the logarithm is a strictly increasing function, this provides a lower bound on $S(f_{1}*f_{2})$ and hence can be used to bound the entropy of f(x,t) of the form in (22). In Section 3 lower bounds on $\dot{S}$ will be derived.

It should be noted that (24) and (25) only apply for convolution on Euclidean spaces, and do not even apply for diffusion processes on the circle. In contrast, other bounds on non-Euclidean spaces and for processes that are not necessarily homogenoues diffusions are presented later in this paper.

### 2.3. Examples

#### 2.3.1. Brownian Motion in Euclidean Spaces

Brownian motion in *d*-dimensional Euclidean space with Dirac delta initial conditions was already reviewed, with pdf (10) and entropy (11). From this, the rate of entropy production can be computed explicitly as:

$$\dot{S}\;=\;\frac{\left(d/2\right){\left(2\pi e\right)}^{d/2}{\left|D\right|}^{1/2}t^{d/2-1}}{{\left(2\pi e\right)}^{d/2}{\left|D\right|}^{1/2}t^{d/2}}=\frac{d}{2t}\;.$$

The version of the Fisher information matrix in the theorem for a Gaussian is the inverse of the covariance, and hence:

$$F\;=\;{\left(Dt\right)}^{-1}=t^{-1}D^{-1}\;.$$

Consequently, (20) gives:

$$\dot{S}\;=\;\frac{1}{2}\mathrm{trace}\left(t^{-1}I\right)=\frac{d}{2t}\;.$$

#### 2.3.2. Brownian Motion on the Torus/Circle

The stochatic differential equation

$$d\theta =\sqrt{D}dw$$

with constant scalar *D* describing Brownian motion on the unit circle has an associated Fokker–Planck equation

$$\frac{\partial f}{\partial t}=\frac{1}{2}D\frac{\partial^{2}f}{\partial \theta^{2}}$$

which is the same as the case on the line. However, the boundary condition θ(π)=θ(−π) is imposed rather than free boundary conditions.

Let:

$$\rho \left(x,t\right)\;≐\;\frac{1}{\sqrt{2\pi Dt}}e^{-\;\frac{x^{2}}{2Dt}}\;,$$

which is the solution of the heat equation on the real line subject to initial conditions ρ(x,0)=δ(x).

The solution of the heat equation on the circle subject to initial condition f(θ,0)=δ(θ) is then [3,5,6]

(26) $$f\left(\theta ,t\right)\;=\;\sum_{k=-\infty }^{\infty }\rho \left(\theta -2\pi k;t\right)=\frac{1}{2\pi }+\frac{1}{\pi }\sum_{n=1}^{\infty }e^{-Dtn^{2}/2}\cos n\theta$$

The above two equalities represent two very different ways of describing the solution. In the first equality, the Gaussian solution presented in the previous section is wrapped (or folded) around the circle. The second solution is a Fourier series solution. The first is efficient when kt is small. In such cases only k=0 may be sufficient as an approximation. When kt is large, truncating n=1 in the Fourier expansion may be sufficient.

Statistical quantities such as the mean and variance can be computed in closed form as:

$$\mu \left(t\right)\;=\;\int_{-\pi }^{\pi }\theta f\left(\theta ,t\right)d\theta \;=\;0$$

and

$$\sigma^{2}\left(t\right)\;=\;\int_{-\pi }^{\pi }\theta^{2}f\left(\theta ,t\right)d\theta \;=\;\frac{\pi^{2}}{3}\;+\;4\sum_{n=1}^{\infty }\frac{{\left(-1\right)}^{n}}{n^{2}}e^{-Dtn^{2}/2}\;.$$

In both expressions in (26) summations are present which makes the exact analytical computation of logarithms, and hence entropy, problematic. However, Since the function Φ(x)=−logx is a monotonically decreasing function, then when a,b>0 we have Φ(a+b)<Φ(a). Consequently, since ρ>0 everywhere,

(27) $$S\left(t\right)\;\leq \;-\int_{-\pi }^{\pi }f\left(\theta ,t\right)\log\rho \left(\theta ,t\right)d\theta =\frac{1}{2}\log\left(2\pi Dt\right)+\frac{\sigma^{2}\left(t\right)}{2Dt}\;.$$

In the extreme case when the distribution reaches equilibrium $f_{\infty }\left(\theta \right)=1/\left(2\pi \right)$, this is the maximum entropy possible, and hence:

(28) $$S\left(t\right)\;\leq \;S_{\infty }=\log\left(2\pi \right)\;.$$

It should be noted that all calculations here are done relative to the measure dθ. For a compact space like the circle, it is common to normalize the measure so that:

$$V=\int_{V}\;1\;dV=1.$$

In doing so for the heat kernel on the circle this would involve redefining dV=dθ/2π and $f^{\prime }\left(\theta ,t\right)≐2\pi f\left(\theta ,t\right)$. This has no effect on mean and covariance, but the value of entropy is shifted such that the entropy of the uniform distribution is equal to zero and the entropy of all other distributions are negative. Rewriting (28) in a way that does not depend on the choice of normalization of measure is:

(29) $$S\left(t\right)\;\leq \;\log V\left(S^{1}\right)\;.$$

In other words, the value of entropy not only depends on the base of the logarithm, but also on the way that the integration measure is scaled.

#### 2.3.3. Concentration of Species Transport in Inhomogeneous Compressible Flow

The concentration c(x,t) of a species in inhomogeneous compressible flow can be modeled by the Equation [19,20,21]:

(30) $$\frac{\partial c}{\partial t}\;=\;\frac{\partial }{\partial x}\left[D\left(x,t\right)\frac{\partial c}{\partial x}\right]-\frac{\partial }{\partial x}\left(u(x,t)c\right)$$

where $D\left(x,t\right)=D_{0}{\left(1+\kappa_{0}x\right)}^{2}$ and $u\left(x,t\right)=u_{0}\left(1+\kappa_{0}x\right)$. This is a FPE, and it is possible to work backwards to find the corresponding SDE.

We see immediately from the above partial differential Equation (pde) that if c(x,0) is normalized to be a probability density function, then c(x,t) will preserve this property because integrating both sides over *x* gives that the right side is zero, and the time derivative on the left side commutes with the integral over *x*.

Equation (30) represents a one-dimensional example of what was presented in the theorem, and the rate of entropy increase is:

$$\dot{S}\;=\;\int_{-\infty }^{\infty }\frac{1}{c}D\left(x,t\right){\left(\frac{\partial c}{\partial x}\right)}^{2}dx+u_{0}\kappa_{0}$$

since the drift term in the entropy rate computation simplifies as:

$$-\int_{-\infty }^{\infty }u\frac{\partial c}{\partial x}dx\;=\;\int_{-\infty }^{\infty }c\frac{\partial u}{\partial x}dx=u_{0}\kappa_{0}\int_{-\infty }^{\infty }c\left(x,t\right)dx=u_{0}\kappa_{0}\;.$$

#### 2.3.4. Homogeneous Transport in Couette Flow

As another example from classical fluid mechanics, consider 2D homogeneous transport in Couette flow governed by Equation [22,23]:

$$\frac{\partial c}{\partial t}\;=\;D_{0}\left[\frac{\partial^{2}c}{\partial x^{2}}+\frac{\partial^{2}c}{\partial y^{2}}\right]-\frac{U_{0}}{H}y\frac{\partial c}{\partial x}$$

where y∈[0,H] and again c(x,0) is normalized to be a probability density function, and hence c(x,t) retains this property. In this case the region over which the equation holds is an infinite slab with the concentration and its gradient vanishing at y=0 and y=H.

We then arrive at:

$$\dot{S}\;=\;D_{0}\int_{0}^{H}\int_{-\infty }^{\infty }\frac{1}{c}\left[{\left(\frac{\partial c}{\partial x}\right)}^{2}+{\left(\frac{\partial c}{\partial y}\right)}^{2}\right]dxdy+\mu \;\cdot e_{2}$$

where

$$\mu \;\;=\;\int_{0}^{H}\int_{-\infty }^{\infty }x\;c\left(x,t\right)dx$$

is the mean of c(x,t).

The pdf for concentration in both of the above examples can be solved in closed form using the methods in [23] in which inhomogeneous processes on Euclidean space are recast as homogeneous processes on an appropriately chosen Lie group. However, the purpose of these examples is to illustrate the relationship between entropy rate and Fisher information. In the next section, a classical result of information theory is used to bound the Fisher information with covariance. Covariance can be propagated without explicit knowledge of the pdf, which will be demonstrated.

## 3. Bounding Rate of Entropy Production with Covariance

The version of the Fisher information matrix that appears in entropy rate computations in general is not easy to compute. An exception to this statement is when the pdf is a Gaussian and

$$F_{gauss}\;=\;\Sigma_{gauss}^{-1}\;.$$

For other exponential families closed form expressions are also possible. However, in general computing the time-varying Fisher information matrix for a pdf satisfying a Fokker–Planck equation will not be easy to compute.

However, it is possible to bound the Fisher information matrix with the covariance of the pdf, and covariance can be propagated as an ordinary differential equation even when no explicit solution for the time-varying pdf is known.

In this section, the Cramér-Rao Bound [24,25], a famous inequality in Euclidean statistics and information theory, is reviewed. Recently it has been extended to manifolds and Lie groups [26,27,28,29,30,31]. A second kind of inequality for compact spaces such as tori, spheres, and rotation groups was introduced in [32]. Then examples where covariance is propagated directly from the FPE are given in which the aforementioned inequalities can be put to use in bounding the rate of entropy generation. An outline of the general procedure is given here.

Let μ and Σ respectively denote the mean and covariance of a pdf f(x;μ,Σ). Then:

$$\mu \;\;≐\;\int_{R^{n}}x\;f\left(x;\mu \;,\Sigma \right)\;dx\;,$$

or equivalently

(31) $$\int_{R^{n}}\left(x-\mu \;\right)\;f\left(x;\mu \;,\Sigma \right)\;dx\;=\;0\;,$$

and

$$\Sigma \;≐\;\int_{R^{n}}\left(x-\mu \;\right){\left(x-\mu \;\right)}^{T}\;f\left(x;\mu \;,\Sigma \right)\;dx\;.$$

When presented with a FPE of the form:

$$\frac{\partial f}{\partial t}\;=\;Df\;,$$

where D is as in the right hand side of (16), an ordinary differential equation describing the evolution of μ(t) and Σ(t) can be defined as:

$$\dot{\mu \;}=\int_{R^{n}}x\;Df\;dx$$

and

$$\dot{\Sigma }=\int_{R^{n}}\left(x-\mu \;\right){\left(x-\mu \;\right)}^{T}Df\;dx$$

where integration by parts can be used in some cases to obtain explicit closed-form expressions for the integrals on the right hand side of both equations. Once Σ(t) is obtained, it can be used to bound entropy from above using (gaussianentropy) the rate of entropy production from below using the results given in the next section.

### 3.1. The Cramér-Rao Bound

The Cramér-Rao Bound (or CRB) is a way to bound the covariance of an estimated statistical quantity [24,25]. Here it will not be used in its most general form. Here it will only be used in the unbiased estimation of the mean of a probability density function on $R^{n}$.

In the standard derivation of the CRB, as given in [18,33] for the case of estimation of the mean, the gradient of this expression with respect to μ is computed, where ∂f/∂μ denotes the gradient as a column vector, and $\partial f/\partial \mu {\;}^{T}≐{\left[\partial f/\partial \mu \;\right]}^{T}$.

Differentiation of both sides of (31) with respect to $\mu {\;}^{T}$ gives:

$$\frac{\partial }{\partial \mu {\;}^{T}}\int_{R^{n}}\left[x-\mu \;\right]\;f\;dx=\int_{R^{n}}\left[x-\mu \;\right]\frac{\partial \;f\;}{\partial \mu {\;}^{T}}dx-I=O\;.$$

Here the derivative is taken under the integral. The product rule for differentiation then is used with the fact that *f* is a pdf in x. O denotes the m×m zero matrix resulting from computing $\partial 0/\partial \mu {\;}^{T}$. That is, since the zero vector is a constant quantity, each partial derivative with respect to $\mu_{i}$ is zero as well, resulting in an array of zero vectors, O.

The above equation can be written as [18,24,25]

(32) $$I=\int_{R^{n}}a\left(x,\mu \;\right)b^{T}\left(x,\mu \;\right)dx\in R^{p\times m}$$

where

$$a\left(x,\mu \;\right)={\left[\;f\;\right]}^{\frac{1}{2}}\left[x-\mu \;\right]$$

and

$$b\left(x,\mu \;\right)={\left[\;f\;\right]}^{-\frac{1}{2}}\frac{\partial f}{\partial \mu \;}\;.$$

Using the fact that f(x;μ,Σ)=f(x−μ;0,Σ) means that:

$$b\left(x,\mu \;\right)=-{\left[\;f\;\right]}^{-\frac{1}{2}}\frac{\partial f}{\partial x}\;.$$

Then it becomes clear that:

(33) $$F\;=\;\int_{R^{n}}b\left(x,\mu \;\right){\left[b\left(x,\mu \;\right)\right]}^{T}dx$$

and

(34) $$\Sigma \;=\;\int_{R^{n}}a\left(x,\mu \;\right){\left[a\left(x,\mu \;\right)\right]}^{T}dx.$$

Following the logic in [33], two arbitrary vectors are introduced: $v\in R^{p}$ and $w\in R^{m}$. Then (32) is multiplied on the left by $v^{T}$ and on the right by w to give:

(35) $$v^{T}I\;w=\int_{R^{n}}v^{T}a\left(x,\mu \;\right)b^{T}\left(x,\mu \;\right)w\;dx.$$

Regrouping terms in the resulting expression, squaring, and using the Cauchy–Schwarz inequality, then gives [24,25,33]:

$$\begin{array}{ccc} {\left(\int_{R^{n}}v^{T}\left(ab^{T}\right)w\;dx\right)}^{2} & = & {\left(\int_{R^{n}}\left(v^{T}a\right)\left(b^{T}w\right)dx\right)}^{2}\\ & \leq & \left(\int_{R^{n}}{\left(v^{T}a\right)}^{2}dx\right)\left(\int_{R^{n}}{\left(w^{T}b\right)}^{2}dx\right)\\ & = & \left(\int_{R^{n}}v^{T}aa^{T}v\;dx\right)\left(\int_{R^{n}}w^{T}bb^{T}w\;dx\right). \end{array}$$

From the Equations (33)–(35), this can be written as:

$${\left(v^{T}I\;w\right)}^{2}\leq \left(v^{T}\Sigma v\right)\left(w^{T}Fw\right).$$

Making the choice of $w=F^{-1}v$ yields:

$${\left(v^{T}\;F^{-1}v\right)}^{2}\leq \left(v^{T}\Sigma v\right)\left(v^{T}\;F^{-1}v\right).$$

This simplifies to:

(36) $$v^{T}\left(\Sigma -\;F^{-1}\right)v\geq 0\;\;\mathrm{for}\;\mathrm{arbitrary}\;\;v\in R^{n}$$

Consequently, the term in parenthesis is a positive definite matrix, or as a matrix inequality [18,24,25,33]:

(37) $$\Sigma \geq \;F^{-1}\;,$$

which is the famous Cramér-Rao Bound (for the special case of an unbiased estimator of the mean). This is equivalently:

(38) $$\Sigma^{-1}\leq \;F\;.$$

Then, for example, in all of the equations for entropy production in time-varying pdfs on Euclidean space presented earlier, it is possible to bound from below using a cascade of inequalities such as:

$$\mathrm{tr}\left[DF\right]\;\geq \;\lambda_{min}\left(D\right)\;\mathrm{tr}\left[F\right]\;\geq \;\lambda_{min}\left(D\right)\;\mathrm{tr}\left[\Sigma^{-1}\right]\;.$$

### 3.2. An Example

Returning to the example of species transport in a compressible 1D flow outlined in Section 2.3.3, this section illustrates how the entropy rate can be bounded from below using the CRB even when a closed-form solution for the pdf is not known.

From the FPE itself, it is possible to propagate the mean and covariance. Multiplying both sides of (30) and integrating by parts gives the following ordinary differential Equation (ODE) for the mean μ(t)

$$\dot{\mu }\;=\;\left(2d_{0}\kappa_{0}+u_{0}\right)\left(1+\kappa_{0}\mu \right)$$

subject to initial conditions, $\mu \left(0\right)=\mu_{0}$. This ODF can be solved in closed form for μ(t). However, even if it could not be, it could be solved by numerical integration, which is much easier than solving the FPE. Similarly, since:

$$\sigma^{2}=\int_{-\infty }^{\infty }{\left(x-\mu \right)}^{2}c\left(x,t\right)dx=-\mu^{2}+\int_{-\infty }^{\infty }x^{2}c\left(x,t\right)dx\;,$$

multiplying (30) by $x^{2}$ and integrating by parts gives a way to propagate the covariance with an ODE of the form:

$$\frac{d}{dt}\left(\sigma^{2}\right)=F\left(\mu ,\sigma^{2}\right)$$

which can be solved either analytically or numerically subject to initial conditions $\sigma^{2}\left(0\right)=\sigma_{0}^{2}$.

It is worth noting that even in cases where such propagation of moments by FPE is not possible (for example, when higher moments creep into the equations so that there is not form closure), it is still possible to numerically generate a large ensemble of sample paths from the SDE corresponding to the FPE and compute variance (or covariance in multi-dof systems). Covariance estimation is much more stable than pdf estimation, and so using the CRB as a lower bound is more reliable than directly attempting to compute entropy, entropy rate, or Fisher information when the pdf is not known explicitly.

## 4. Classical Statistical Mechanics as Stochastic Mechanics

Classical statistical mechanics, as developed by Boltzmann, Maxwell, and Gibbs, states that entropy increases. For an introduction to phase space and equilibrium statistical mechanics see [34]. Nonequilibrium statistical mechanics has been studied extensively over a long period of time starting with Boltzmann and summarized in a number of books including [35,36,37,38,39]. Important results continue to be developed in modern time, e.g., [40]. An alternative to the classical Boltzmann–Gibbs formulation is stochastic mechanics [41,42,43,44]. The difference is that in Boltzmann’s original formulation of statistical mechanics the model describing collisions between gas molecules was deterministic. At the beginnning of the twentieth century Einstein’s formulation of Brownian motion also did not explicitly model random forces, though Langevin did. In all of those early works on Brownian motion there was no concept of Wiener process or Itô or Stratonovich stochastic calculus. These are mid twentieth century constructs that came after. Consequently, revisiting results in statistical mechanics using more modern stochastic modeling techniques sheds light on some old problems and provides a basis for building connections between statistical mechanics, stochastic modeling, and information theory.

Here a Hamiltonian formulation of stochastic mechanics is used. The Hamiltonian of a mechanical system is defined as the total system energy written in terms the conjugate momenta p and generalized coordinates q:

(39) $$H\left(p,q\right)≐\frac{1}{2}p^{T}M^{-1}\left(q\right)\;p\;+\;V\left(q\right)\;.$$

Here M(q) is the configuration-dependent mass matrix and

$$p\;≐\;M\left(q\right)\dot{q}\;.$$

The beauty of the Hamiltonian formulation is that the volume in phase space (i.e., the joint p-q space) is invariant under coordinate changes.

### 4.1. Properties of Phase Space

As is well known and explained in [5,6], if q and $q^{\prime }$ are two different sets of coordinates, then kinetic energy is expressed as:

$$T\;=\;\frac{1}{2}{\dot{q}}^{\prime }{}^{T}M^{\prime }\left(q^{\prime }\right){\dot{q}}^{\prime }=\frac{1}{2}{\dot{q}}^{T}M\left(q\right)\dot{q}.$$

Then with the Jaccobian relating rates of change,

$${\dot{q}}^{\prime }\;=\;J\left(q\right)\;\dot{q},$$

it is clear that:

$$M\left(q\right)\;=\;J^{T}\left(q\right)M^{\prime }\left(q^{\prime }\left(q\right)\right)J\left(q\right)\;.$$

From the above, and the definition of conjugate momenta, $p_{i}≐\partial T/\partial {\dot{q}}_{i}$,

(40) $$p^{\prime }=J^{-T}\left(q\right)\;p.$$

Therefore, the two phase spaces have volume elements that are related as:

$$dp^{\prime }\;dq^{\prime }\;=\;\left|\begin{array}{cc} J^{-T}\left(q\right) & \partial (J^{-T}\left(q\right)p)/\partial q\\ O & J(q) \end{array}\right|\;dp\;dq.$$

The determinant of the upper-triangular block matrix in the above equation is equal to 1, illustrating the invariance:

(41) $$dp\;dq\;=\;dp^{\prime }\;dq^{\prime }\;.$$

The key to this result is how p transforms in (40). A similar result holds in the Lie group setting wherein the cotangent bundle of a Lie group can be endowed with an operation making it unimodular even when the underlying group is not [45]. This is analogous to why (4) requires the metric tensor weighting and is coordinate dependent and (41) is not.

### 4.2. Hamilton’s Equations for a System Forced by External Noise and Damping

Hamilton’s equations of motion are:

(42) $$\begin{array}{ccc} \frac{dp_{i}}{dt} & \;=\; & -\frac{\partial H}{\partial q_{i}}+F_{i} \end{array}$$

(43) $$\begin{array}{ccc} \frac{dq_{i}}{dt} & \;=\; & \;\frac{\partial H}{\partial p_{i}}\;. \end{array}$$

where $F_{i}$ are generalized external forces. In the case in which the mechanical system is forced by noise and viscous damping, then after multiplication by dt, these equations of motion become:

(44) $$\begin{array}{c} dp_{i}\;=\;-\frac{1}{2}p^{T}\frac{\partial M}{\partial q_{i}}p\;dt\;-\;\frac{\partial V}{\partial q_{i}}\;dt\;-\;e_{i}^{T}CM^{-1}p\;dt\;+\;e_{i}^{T}Bdw \end{array}$$

and

(45) $$dq_{i}\;=\;e_{i}^{T}M^{-1}p\;dt$$

where $e_{i}$ is the $i^{th}$ natural unit basis vector. Note that the configuration-dependant mass matrix M=M(q), noise matrix B=B(q), and damping C=C(q) appear prominently in these equations.

Equations (44) and (45) can be written together as:

(46) $$\left(\begin{array}{c} dq\\ dp \end{array}\right)=\left(\begin{array}{c} \alpha \;(p,q)\\ \gamma \;(p,q) \end{array}\right)\;dt+\left(\begin{array}{cc} O & O\\ O & B(q) \end{array}\right)\left(\begin{array}{c} dw^{\prime }\\ dw \end{array}\right)\;.$$

(Here $dw^{\prime }$ multiplies zeros and hence is inconsequential).

The vector-valued function α and γ are defined by their entries:

$$\begin{array}{ccc} \alpha_{i} & ≐ & e_{i}^{T}M^{-1}p\\ \gamma_{i} & ≐ & -\frac{1}{2}p^{T}\frac{\partial M}{\partial q_{i}}p-\frac{\partial V}{\partial q_{i}}-e_{i}^{T}CM^{-1}p. \end{array}$$

The Fokker–Planck corresponding to (46), which together with an initial distribution $f\left(q,p,0\right)=f_{0}\left(q,p\right)$ defines the family of time-evolving pdfs f(q,p;t), is

(47) $$\frac{\partial f}{\partial t}+\sum_{i=1}^{n}\frac{\partial }{\partial q_{i}}\left(\alpha_{i}f\right)+\sum_{i=1}^{n}\frac{\partial }{\partial p_{i}}\left(\gamma_{i}f\right)-\frac{1}{2}\sum_{k=1}^{n}\sum_{i,j=1}^{n}\frac{\partial^{2}}{\partial p_{i}\partial p_{j}}\left(b_{ik}b_{kj}^{T}f\right)=0$$

where $b_{ij}=e_{i}^{T}Be_{j}$ is the $i,j^{th}$ entry of *B*.

Note that for any mechanical system with inertia the diffusion is the same regardless of Itô or Stratonovich interepretation, as:

$$\frac{\partial^{2}}{\partial p_{i}\partial p_{j}}\left(b_{ik}b_{kj}^{T}f\right)=\frac{\partial }{\partial p_{i}}\left(b_{ik}\frac{\partial }{\partial p_{j}}\left(b_{kj}^{T}f\right)\right)=b_{ik}b_{kj}^{T}\frac{\partial^{2}f}{\partial p_{i}\partial p_{j}}\;.$$

That is, even though *B* is configuration dependent, the structure of the FPE equations in the case of mechanical systems with inertia places partial derivatives with respect to momenta in the diffusion terms, and such partial derivatives pass through the configuration-dependent *B* matrix. For this reason in mechanical systems with inertia, it does not matter if Itô or Stratonovich interpretations of SDEs are used. This freedom allows the modeler to take the best of both worlds. However, when approximations are made in modeling the initial equations of motion, such as assuming that the inertia is negligible, then the above Hamiltonian formulation no longer applies and one must be very careful as to the interpretation of the SDE as Itô or Stratonovich.

### 4.3. The Boltzmann Distribution

The Boltzmann distribution is defined as:

(48) $$f_{\infty }\left(q,p\right)\;≐\;\frac{1}{Z}\exp\left(-\beta H(p,q)\right)$$

where $\beta ≐1/k_{B}T$ with $k_{B}$ denoting Boltzmann’s constant and *T* is temperature measured in degrees Kelvin.

The *partition function* is defined as:

(49) $$Z=\int_{q}\int_{p}\exp\left(-\beta \;H(p,q)\right)\;dp\;dq.$$

The reason for using the subscript *∞* in defining (48) is the following theorem.

**Theorem** **2.** *If $q\in R^{n}$ globally parameterizes the configuration manifold of a mechanical system, then the solution of the Fokker–Planck Equation (47) will satisfy*

$$\lim_{t\to \infty }f\left(p,q,t\right)\;=\;f_{\infty }\left(p,q\right)$$

*if and only if*

(50) $$C=\frac{\beta }{2}BB^{T}.$$

**Proof.**  We begin by noting that (47) can be simplified a bit. First,

$$\frac{\partial }{\partial q_{i}}\left(\alpha_{i}f\right)\;=\;\frac{\partial \alpha_{i}}{\partial q_{i}}f+\alpha_{i}\frac{\partial f}{\partial q_{i}}$$

and

$$\frac{\partial }{\partial p_{i}}\left(\gamma_{i}f\right)\;=\;\frac{\partial \gamma_{i}}{\partial p_{i}}f+\gamma_{i}\frac{\partial f}{\partial p_{i}}\;.$$

It is not difficult to show that:

$$\sum_{i=1}^{n}\left\{\frac{\partial \alpha_{i}}{\partial q_{i}}\;+\;\frac{\partial \gamma_{i}}{\partial p_{i}}\right\}\;=\;\mathrm{tr}\left(CM^{-1}\right)\;.$$

Using this, and considering the equilibrium condition when ∂f/∂t=0 then reduces (47) to

(51) $$\mathrm{tr}\left(CM^{-1}\right)f+\sum_{i=1}^{n}\left\{\alpha_{i}\frac{\partial f}{\partial q_{i}}\;+\;\gamma_{i}\frac{\partial f}{\partial p_{i}}\right\}-\frac{1}{2}\sum_{i,j=1}^{n}\frac{\partial^{2}}{\partial p_{i}\partial p_{j}}\left({\left(BB^{T}\right)}_{ij}f\right)=0\;,$$

where the substitution

$${\left(BB^{T}\right)}_{ij}\;=\;\sum_{k=1}^{n}b_{ik}b_{kj}^{T}$$

has been made.

Note that:

$$\frac{\partial f_{\infty }}{\partial q_{i}}\;=\;-\beta \left(\frac{\partial V}{\partial q_{i}}+\frac{1}{2}p^{T}\frac{\partial M}{\partial q_{i}}p\right)f_{\infty }\;,$$

$$\frac{\partial f_{\infty }}{\partial p_{i}}\;=\;-\beta e_{i}^{T}M^{-1}p\;f_{\infty }\;=\;-\beta \alpha_{i}\;f_{\infty }\;,$$

and hence significant cancelation results in:

$$\sum_{i=1}^{n}\left\{\alpha_{i}\frac{\partial f_{\infty }}{\partial q_{i}}\;+\;\gamma_{i}\frac{\partial f_{\infty }}{\partial p_{i}}\right\}\;=\;\beta \alpha {\;}^{T}C\alpha \;\;.$$

Moreover,

$$\frac{\partial^{2}f_{\infty }}{\partial p_{i}\partial p_{i}}\;=\;\left(-\beta m_{ij}^{-1}+\beta^{2}\alpha_{i}\alpha_{j}\right)\;f_{\infty }$$

Substituting into (51) therefore gives:

$$\mathrm{tr}\left(CM^{-1}\right)\;+\;\beta \alpha {\;}^{T}C\alpha \;\;-\;\frac{\beta }{2}\mathrm{tr}\left(M^{-1}BB^{T}\right)\;-\;\frac{\beta^{2}}{2}\alpha {\;}^{T}BB^{T}\alpha \;\;=\;0.$$

This shows that $f_{\infty }\left(p,q\right)$ is in fact a solution of (51), if (50) holds. The necessary conditions for the above equality to hold boil down to the necessary conditions for the two independent statements:

$$\mathrm{tr}\left(\left(C\;-\;\frac{\beta }{2}BB^{T}\right)M^{-1}\right)\;=\;0$$

and

$$\alpha {\;}^{T}\left(C\;-\;\frac{\beta }{2}BB^{T}\right)\alpha \;\;=\;0$$

to hold. The independence of these follows from the fact that some terms depend on α and others do not, and the main equality must hold for all values of α.

The only way that both of the above can hold is if $C\;-\;\frac{\beta }{2}BB^{T}$ is skew symmetric. However, damping matrices, like stiffness and mass matrices, are symmetric, as is $BB^{T}$. Hence (50) must hold. □

Note that the necessary and sufficient conditions in (50) for the Boltzmann distribution to be the equilibrium/stationary solution holds even when *B* and *C* are dependant on q. As such, this is a generalization of the fluctuation dissipation theorem (which is stated for particles) to the case of complex mechanical systems that can be modeled as a collection of rigid bodies (e.g., biological macromolecules).

### 4.4. Marginal Densities and the Conundrum as Mass Becomes Zero

Marginal densities of f(p,q,t) can be defined as:

$$f\left(p,t\right)\;≐\;\int_{q}f\left(p,q,t\right)\;{\left|\det M\left(q\right)\right|}^{1/2}\;dq$$

and

(52) $$f\left(q,t\right)\;≐{\;|\det M\left(q\right)|}^{-1/2}\int_{p}f\left(p,q,t\right)\;dp\;,$$

which is consistent with (3).

In the equilibrium case it is always possible to compute $f_{\infty }\left(q\right)$ in closed form as:

(53) $$f_{\infty }\left(q\right)=\frac{1}{Z_{c}}e^{-\beta V(q)}$$

where

$$Z_{c}\;=\;\int_{q}e^{-\beta V(q)}{\left|\det M\left(q\right)\right|}^{1/2}dq$$

is the configurational partition function. Then,

$$\int_{q}f_{\infty }\left(q\right)\;{\left|\det M\left(q\right)\right|}^{1/2}\;dq\;=\;1\;.$$

In contrast, in general $f_{\infty }\left(p\right)$ can only be computed easily in closed form when $M\left(q\right)=M_{0}$ is constant. In this case:

$$f_{\infty }\left(p\right)=\frac{\beta^{d/2}}{{\left(2\pi \right)}^{d/2}{\left|\det M_{0}\right|}^{d/2}}\exp\left(-\frac{\beta }{2}p^{T}M_{0}^{-1}p\right)$$

is a Gaussian distribution in the momenta.

Though (53) degenerates as the inertia goes to zero, it does so gracefully since both ${\left|\det M\right|}^{1/2}$ and $Z_{c}$ approach zero in the same way as the system mass goes to zero. We can then use it as the baseline truth to compare approximations in which inertia is neglected. For example, consider the spring-mass-damper with noise:

$$m\ddot{x}+c\left(x\right)\dot{x}+kx=b\left(x\right)n$$

where c(x) and b(x) are nonlinear functions satisfying the condition $2c\left(x\right)=\beta b{\left(x\right)}^{2}$. If ndt=dw, then as m→0, we have a conundrum unless $c=c_{0}$ and $b=b_{0}$ are constant. Namely, which of the following interpretations is correct?

$$dx_{1}=-k\;c{\left(x_{1}\right)}^{-1}\;x_{1}dt\;+\;2\beta^{-1}b{\left(x_{1}\right)}^{-1}\;dw$$

or

$$dx_{2}=-k\;c{\left(x_{2}\right)}^{-1}\;x_{2}dt\;+\;2\beta^{-1}b{\left(x_{2}\right)}^{-1}\;Ⓢ\;dw\;?$$

It did not matter when there was inertia, as both gave the same FPEs in the case, but making the approximation that the mass is zero creates a situation where a choice now must be made.

The answer can be informed by comparing the corresponding pdfs that solve the associated Fokker–Planck equations, $f_{1}\left(x,t\right)$ and $f_{2}\left(x,t\right)$, with f(x,t) in (52). Short of that, we can examine the behavior of the mean as a function of time, and the behavior of the equilibrium distributions as compared with (52).

The Fokker–Planck equations corresponding to the above SDEs are, respectively:

$$\frac{\partial f_{1}}{\partial t}=k\frac{\partial }{\partial x}\left(c^{-1}xf_{1}\right)+2\beta^{-2}\frac{\partial^{2}}{\partial x^{2}}\left(b^{-2}f_{1}\right)$$

and

$$\frac{\partial f_{2}}{\partial t}=k\frac{\partial }{\partial x}\left(c^{-1}xf_{2}\right)+2\beta^{-2}\frac{\partial }{\partial x}\left(b^{-1}\frac{\partial }{\partial x}\left(b^{-1}f_{2}\right)\right)\;.$$

Expanding and considering equilibrium conditions gives

$$\Delta_{1}=k\frac{\partial }{\partial x}\left(c^{-1}xf_{1}\right)+2\beta^{-2}\frac{\partial }{\partial x}\left(-2b^{-3}\frac{\partial b}{\partial x}f_{1}+b^{-2}\frac{\partial f_{1}}{\partial x}\right)$$

and

$$\Delta_{2}=k\frac{\partial }{\partial x}\left(c^{-1}xf_{2}\right)+2\beta^{-2}\frac{\partial }{\partial x}\left(-b^{-3}\frac{\partial b}{\partial x}f_{2}+b^{-2}\frac{\partial f_{2}}{\partial x}\right)$$

where an exact solution would give $\Delta_{i}=0$.

The exact configurational marginal from the Hamiltonian formulation is

$$f_{\infty }\left(x\right)={\left(\frac{\beta k}{2\pi }\right)}^{\frac{1}{2}}e^{-\beta kx^{2}/2}\;,$$

and it has the property

$$\frac{\partial f_{\infty }}{\partial x}\;=\;-\beta kxf_{\infty }\;.$$

Substituting into the above, and observing that

$$k\frac{\partial }{\partial x}\left(c^{-1}xf_{\infty }\right)+2\beta^{-2}\frac{\partial }{\partial x}\left(b^{-2}\frac{\partial f_{\infty }}{\partial x}\right)\;=\;0$$

due to the relationship between *b* and *c*, then

$$\Delta_{1}=2\beta^{-2}\frac{\partial }{\partial x}\left(-b^{-3}\frac{\partial b}{\partial x}f_{\infty }\right)\;=\;2\Delta_{2}\;.$$

This means that neither interpretation gives the true answer at equilibrium, but the magnitude of the discrepancy in the Stratonovich model is half that of the Itô. For this reason, unless modeling systems in phase space, or if there are physical grounds for choosing a particular SDE (e.g., working backwards from Fick’s Law), it is safest to consider diffusions with constant diffusion tensors, as will be the case throughout the remainder of this paper.

## 5. Stochastic Systems on Unimodular Lie Groups

A stochastic mechanical system that has a Lie group as its configuration space can be studied in a coordinate-free way [5,6]. These systems can be purely kinematic, or can have inertia. Concrete examples are used here to illustrate, and then general theorems are provided to quantify the rate of entropy production. Different connections between Lie groups and thermodynamics than what is presented here have been made in the literature [46,47,48,49].

### 5.1. Review of Unimodular Matrix Lie Groups with SO(3) and SE(2) as Examples

The use of geometric (and particularly Lie-theoretic) methods in the control of mechanical systems and robots has been studied extensively over the past half century [50,51,52,53,54]. The material and notation in this section summarizes more in-depth treatments in [3,5,6].

A matrix Lie group is a group with elements that are matrices, for which group multiplication is matrix multiplication, and for which the underlying space is an analytic manifold with the operations of group multiplication and inversion of elements being analytic also. Intuitively, matrix Lie groups are continuous families of invertible matrices with structure that is preserved under multiplication and inversion. The dimension of a matrix Lie group is the dimension of its manifold, not the dimension of the square matrices describing its elements.

For example, the group of rigid-body displacements in the Euclidean plane, SE(2), can be described with elements of the form:

(54) $$g\left(x,y,\theta \right)\;=\;\left(\begin{array}{ccc} \cos\theta & -\sin\theta & x\\ \sin\theta & \cos\theta & y\\ 0 & 0 & 1 \end{array}\right)\;.$$

The dimension is 3 because there are three free parameters (x,y,θ). This group is not compact as *x* and *y* can take values on the real line.

The group of pure rotations in 3D can be described by the rotation matrices:

$$SO\left(3\right)\;≐\;\left\{R\in R^{3\times 3}\;|\;RR^{T}=I\;,\;\det R=+1\right\}\;.$$

SO(3) is a compact 3-dimensional manifold. Again, the fact that the dimension of the matrices is also 3 is coincidental.

A unimodular Lie group is defined by the property that a measure dg can be constructed such that the integral over the group has the property that

(55) $$\int_{G}f\left(g\right)\;dg\;=\;\int_{G}f\left(g_{0}g\right)\;dg\;=\;\int_{G}f\left(gg_{0}\right)\;dg$$

for any fixed $g_{0}\in G$ and any function $f\in L^{1}\left(G\right)$. It can also be shown that as a consequence of (55):

(56) $$\int_{G}f\left(g\right)\;dg\;=\;\int_{G}f\left(g^{-1}\right)\;dg\;.$$

These properties are natural generalizations of those familiar to us for functions on Euclidean space.

As we are primarily concerned with probability density functions for which

$$\int_{G}f\left(g\right)dg=1\;,$$

these clearly meet the condition of being in $L^{1}\left(G\right)$.

In the case of SO(3) the bi-invariant measure expressed in terms of Z−X−Z Euler angles (α,β,γ) is dR=sinβdαdβdγ. In the case of SE(2), the bi-invariant measure is dg=dxdydθ.

The convolution of probability density functions on a unimodular Lie group is a natural operation, and is defined as:

(57) $$\left(f_{1}*f_{2}\right)\left(g\right)\;≐\;\int_{G}f_{1}\left(h\right)f_{2}\left(h^{-1}g\right)\;dh\;.$$

The convolution of two probability density functions is again a probability density.

In addition to being natural spaces over which to integrate probability density functions, natural concepts of directional derivatives of functions exist in the matrix Lie group setting. This builds on the fact that associated with every matrix Lie group is a matrix Lie algebra.

In the case of SO(3), the Lie algebra consists of 3×3 skew-symmetric matrices of the form

(58) $$X=\left(\begin{array}{ccc} 0 & -x_{3} & x_{2}\\ x_{3} & 0 & -x_{1}\\ -x_{2} & x_{1} & 0 \end{array}\right)=\sum_{i=1}^{3}x_{i}E_{i}.$$

The matrices $\left\{E_{i}\right\}$ form a basis for the set of 3×3 skew-symmetric matrices. The coefficients $\left\{x_{i}\right\}$ are all real. The notation relating the matrix *X* and the vector $x={\left[x_{1},x_{2},x_{3}\right]}^{T}$ is [51,52]

(59) $$x\;=\;X^{\vee }\;\;\;\mathrm{and}\;\;\;X=\hat{x}\;.$$

This is equivalent to identifying $E_{i}^{\vee }$ with $e_{i}$.

For SE(2) the basis elements are different, and are of the form

$$E_{1}^{\prime }=\left(\begin{array}{ccc} 0 & 0 & 1\\ 0 & 0 & 0\\ 0 & 0 & 0 \end{array}\right);\;E_{2}^{\prime }=\left(\begin{array}{ccc} 0 & 0 & 0\\ 0 & 0 & 1\\ 0 & 0 & 0 \end{array}\right);\;E_{3}^{\prime }=\left(\begin{array}{ccc} 0 & -1 & 0\\ 1 & 0 & 0\\ 0 & 0 & 0 \end{array}\right)\;.$$

Every element of the Lie algebra associated with SE(2) can be written as a linear combination of these, and the notation (59) is still used to identify these matrices with natural unit basis vectors $e_{i}\in R^{3}$. For example, $X^{\prime }=x_{1}^{\prime }E_{1}^{\prime }+x_{2}^{\prime }E_{2}^{\prime }+x_{3}^{\prime }E_{3}^{\prime }$. (Here the primes are used so as not to confuse the Lie algebra elements for SE(2) with those for SO(3), but when working with a single Lie group and Lie algebra the primes will be dropped, as in the discussion below which is for the generic case.)

For an arbitrary unimodular matrix Lie group, a natural concept of directional derivative is

(60) $$\left(\tilde{X}f\right)\left(g\right)\;≐\;{\left.\frac{d}{dt}f\left(g\;\exp\left(tX\right)\right)\right|}_{t=0}\;.$$

Here the argument of the function *f* is read as the product of *g* and exp(tX), which are each in *G*, as is the product. If $X=\sum_{i}x_{i}E_{i}$ for constants $\left\{x_{i}\right\}$, this derivative has the property

$$\left(\tilde{X}f\right)\left(g\right)=\sum_{i}x_{i}\left({\tilde{E}}_{i}f\right)\left(g\right)\;.$$

Such derivatives appear in invariant statements of Fokker–Planck equations on unimodular Lie groups. Moreover, these derivatives can be used together with integration to state results such as integration by parts

$$\int_{G}f_{1}\left(g\right)\left({\tilde{E}}_{i}f_{2}\right)\left(g\right)dg\;=\;-\int_{G}f_{2}\left(g\right)\left({\tilde{E}}_{i}f_{1}\right)\left(g\right)dg\;.$$

There are no surface terms because either group is infinite in its extent (and so the functions must decay to zero at the boundaries), or it is compact (in which case the functions must match values when arriving from different directions), or both for a group such as SE(2).

### 5.2. The Noisy Kinematic Cart

The stochastic kinematic cart has been studied extensively in the robotics literature [5,6,55,56,57,58]. In this model (which is like a motor-driven wheelchair) the two wheels each have radii *r*, and the wheelbase (distance between wheels) is denoted as *L*. The nonholonomic equations of motion are

(61) $$\left(\begin{array}{c} \dot{x}\\ \dot{y}\\ \dot{\theta } \end{array}\right)\;=\;\left(\begin{array}{cc} \frac{r}{2}\cos\theta & \frac{r}{2}\cos\theta \\ \frac{r}{2}\sin\theta & \frac{r}{2}\sin\theta \\ \frac{r}{L} & -\frac{r}{L} \end{array}\right)\left(\begin{array}{c} {\dot{\phi }}_{1}\\ {\dot{\phi }}_{2} \end{array}\right).$$

When the wheel rates consist of a constant deterministic part and a stochastic part, then

(62) $$\begin{array}{ccc} d\phi_{1}\; & = & \;\omega \;dt+\sqrt{D}\;dw_{1} \end{array}$$

(63) $$\begin{array}{ccc} d\phi_{2}\; & = & \;\omega \;dt+\sqrt{D}\;dw_{2} \end{array}$$

and multiplying (61) by dt and substituting in (63) results in an SDE. This is an example where it does not matter whether the SDE is of Itô or Stratonovich type, even though *B* is not constant. The corresponding Fokker–Planck equation for the probability density function f(x,y,θ;t) with respect to measure dxdydθ is [58]:

$$\frac{\partial f}{\partial t}=-r\omega \cos\theta \frac{\partial f}{\partial x}-r\omega \sin\theta \frac{\partial f}{\partial y}+$$

$$\frac{D}{2}\left(\frac{r^{2}}{2}\cos^{2}\theta \frac{\partial^{2}f}{\partial x^{2}}+\frac{r^{2}}{2}\sin2\theta \frac{\partial^{2}f}{\partial x\partial y}+\frac{r^{2}}{2}\sin^{2}\theta \frac{\partial^{2}f}{\partial y^{2}}+\frac{2r^{2}}{L^{2}}\frac{\partial^{2}f}{\partial \theta^{2}}\right),$$

which is subject to the initial conditions f(x,y,θ;0)=δ(x−0)δ(y−0)δ(θ−0).

The coordinates (x,y,θ) that define the position and orientation of the cart relative to the world frame are really parameterizing the group of rigid-body motions of the plane, SE(2). Each element of this unimodular Lie group can be described as homogeneous transformation matrices of the form in (54) in which case the group law is matrix multiplication.

Then (61) can be written in coordinate-free notation as:

(64) $${\left(g^{-1}\frac{dg}{dt}\right)}^{\vee }\;=\;A\;\dot{\phi \;}\;\;\;\;\mathrm{where}\;\;\;\;A\;=\;\frac{r}{2}\left(\begin{array}{cc} 1 & 1\\ 0 & 0\\ 2/L & -2/L \end{array}\right)\;.$$

Here the notation ∨ is used as in [3,5,6,51] in analogy with (59), but for the case of SE(2) rather than SO(3).

The coordinate-free version of the above Fokker–Planck equation can be written compactly in terms of these Lie derivatives as [58]:

(65) $$\frac{\partial f}{\partial t}=-r\omega {\tilde{E}}_{1}^{\prime }f+\frac{r^{2}D}{4}{\left({\tilde{E}}_{1}^{\prime }\right)}^{2}f+\frac{r^{2}D}{L^{2}}{\left({\tilde{E}}_{3}^{\prime }\right)}^{2}f$$

with initial conditions f(g;0)=δ(g). The resulting time-evolving pdf is denoted as f(g;t) with respect to the natural bi-invariant integration measure for SE(2), which is dg=dxdydθ. Solutions for (65) can be obtained in different regimes (small Dt and large Dt) either using Lie-group Gaussian distributions or Lie-group Fourier expansions, as in [58,59]. That is not the goal here. Instead, the purpose of this example is to provide a concrete case for the derivations that follow regarding rate of entropy production.

It should be noted that degenerate diffusions on SE(2) occur not only in this problem, in models of the visual cortex [60,61,62,63,64,65]. Phase noise is a problem in coherent optical communication systems that has been identified in the literature [66,67,68,69,70]. The Fokker–Planck equations describing phase noise have been developed and solved using various methods [71,72,73]. Remarkably, these FPEs are the same kind as those for the kinematic cart, inpainting, visual cortex modeling, etc. Moreover, the natural extension of (64) and (65) to SE(3) has found applications in modeling DNA (as reviewed in [3,5,6]) and flexible steerable needles for robotic surgery [57,74,75,76].

### 5.3. Rotational Brownian Motion

Starting with Perrin [77], various efforts at mathematical modeling of rotational Brownian motion has been undertaken over the past century [78,79,80,81,82]. These include both inertial and noninertial theories. A major application is in the spectroscopy of macromolecules [83,84]. Essentially the same mathematics is applicable to modeling the time-evolving uncertaintly in mechanical gyroscopes [85].

Brownian motion on Riemannian manifolds and Lie groups also has been studied over a long period of time in the mathematics literature [86,87,88,89,90,91], with the rotation group and three-sphere being two very popular objects [92,93,94]. In addition to forcing by white noise, forcing by Lévy processes (white noise with jumps) has also been investigated [95].

#### 5.3.1. Inertial Theory

Euler’s equation of motion for a rotating rigid body subjected to an external potential, noise, and damping can be written as:

(66) $$I_{0}\;d\omega \;+\omega \;\times \left(I_{0}\omega \;\right)\;dt=-\left(\tilde{E}V\right)\left(R\right)dt-C_{0}\omega \;dt+B_{0}dw$$

where

$$\left(\tilde{E}V\right)\left(R\right)\;=\;\left(\begin{array}{c} \left({\tilde{E}}_{1}V\right)\left(R\right)\\ \left({\tilde{E}}_{2}V\right)\left(R\right)\\ \left({\tilde{E}}_{3}V\right)\left(R\right) \end{array}\right)\;.$$

Here ω is the body-fixed description of angular velocity, which is related to a time-evolving rotation matrix (using the hat notation in (59)) as:

(67) $$\dot{R}=R\hat{\omega \;}\;.$$

The moment of inertia matrix, $I_{0}$, damping matrix, $C_{0}$, and noise matrix $B_{0}$ are all constant. Equations (66) and (67) define a stochastic process evolving on the tangent bundle of SO(3).

This can be re-written using angular momentum, $\pi \;=I_{0}\omega \;$, as

(68) $$d\pi \;\;=\;\pi \;\times \left(I_{0}^{-1}\pi \;\right)\;dt-\left(\tilde{E}V\right)\left(R\right)dt-C_{0}I_{0}^{-1}\pi \;dt+B_{0}dw\;.$$

Equations (68) and

$$\dot{R}=R\;\hat{I_{0}^{-1}\pi \;}$$

define a stochastic process on the cotangent bundle of SO(3).

Note that p≠π. To see this, expand angular velocity and kinetic energy in coordinates as $\omega \;=J\left(q\right)\dot{q}$ and

$$T=\frac{1}{2}{\dot{q}}^{T}J{\left(q\right)}^{T}I_{0}J\left(q\right)\dot{q}\;.$$

Consequently $M\left(q\right)=J{\left(q\right)}^{T}I_{0}J\left(q\right)$ and $p=M\left(q\right)\dot{q}$. In contrast, $\pi \;=I_{0}J\left(q\right)\dot{q}$. Therefore, in order to use the general results from statistical mechanics, the interconversion

$$p\;=\;J{\left(q\right)}^{T}\pi \;$$

must be done. Moreover, $C_{0}$ in the above equations is not the same as *C* in the Hamiltonian formulation. A Rayleigh dissipation function will be of the form

$$R=\frac{1}{2}\omega {\;}^{T}C_{0}\omega \;=\frac{1}{2}{\dot{q}}^{T}J{\left(q\right)}^{T}C_{0}J\left(q\right)\dot{q}\;,$$

indicating that $C\left(q\right)=J{\left(q\right)}^{T}C_{0}J\left(q\right)$. Then converting (68) to the Hamiltonian form, the viscous and noise terms become:

$$J{\left(q\right)}^{T}\left[-C_{0}I_{0}^{-1}\pi \;dt+B_{0}dw\right]=-J{\left(q\right)}^{T}C_{0}I_{0}^{-1}I_{0}J\left(q\right)dq+J{\left(q\right)}^{T}B_{0}dw\;.$$

If $C\left(q\right)=J{\left(q\right)}^{T}C_{0}J\left(q\right)$ and $B\left(q\right)=J{\left(q\right)}^{T}B_{0}$, and if *J* is invertible, then the condition in (50) then becomes completely equivalent to:

(69) $$C_{0}=\frac{\beta }{2}B_{0}B_{0}^{T}$$

The structure of the $C_{0}$ matrix for a rigid body is a function of its shape. For example, the viscous drag on an ellipsoid was characterized in [96]. Given $C_{0}$, it is possible to define $B_{0}=C_{0}^{1/2}$.

When I=I and V=0 (68) becomes

(70) $$d\pi \;\;=\;-\frac{\beta }{2}B_{0}B_{0}^{T}\pi \;dt+B_{0}dw\;.$$

This is an Ornstein-Uhlenbeck process, and the corresponding Fokker–Planck equation can be solved for f(π,t) in closed form as a time-varying Gaussian if the initial conditions are f(π,0)=δ(π). The equilibrium solution is the Boltzmann distribution

$$f_{\infty }\left(\pi \;\right)=c\left(\beta \right)\exp\left(-\frac{\beta }{2}{∥\pi \;∥}^{2}\right)$$

where c(β) is the usual normalizing constant for a Gaussian distribution.

#### 5.3.2. Noninertial Theory

When the inertia is negligible, as it is in the case of rotational Brownian motion of molecules, then (69) and (66) give

(71) $$\omega \;dt\;=\;B_{1}dw\;\;\;\mathrm{where}\;\;\;B_{1}=\frac{2}{\beta }B_{0}^{-T}\;.\;.$$

This can be expressed in coordinates as a Stratonovich equation:

(72) $$\dot{q}=J^{-1}\left(q\right)B_{1}w\;,$$

or it can be kept in the invariant form (71). The corresponding Fokker–Planck equation is of the form:

$$\frac{\partial f}{\partial t}=\sum_{i,j=1}^{3}D_{ij}{\tilde{E}}_{i}{\tilde{E}}_{j}f$$

where $D=B_{1}B_{1}^{T}$ and each ${\tilde{E}}_{i}$ is as in (60) with $X=E_{i}$.

The short-time solution of this equation subject to Dirac delta initial conditions is the Gaussian in exponential coordinates. Hence, for short times, entropy and entropy rate can be computed in closed form using the results from the Euclidean case.

In noninertial theory a special case is isotropic diffusions. Let:

$$\nabla^{2}\;≐\;{\tilde{E}}_{1}^{2}+{\tilde{E}}_{2}^{2}+{\tilde{E}}_{3}^{2}\;.$$

An isotropic driftless diffusion on SO(3) is one of the form

(73) $$\frac{\partial f}{\partial t}=K\nabla^{2}f\;.$$

The heat kernel for SO(3) is the solution of this subject to the initial condition f(R,0)=δ(R).

Rotation matrices can be expressed in terms of the axis and angle of rotation using Euler’s formula:

$$R\left(\theta ,\nu ,\lambda \right)\;=\;\exp\left[\theta \hat{n}\left(\nu ,\lambda \right)\right]\;=\;I+\sin\theta \;\hat{n}\left(\nu ,\lambda \right)+\left(1-\cos\theta \right)\;{\left[\hat{n}\left(\nu ,\lambda \right)\right]}^{2}$$

where $\hat{n}\left(\nu ,\lambda \right)$ is a skew symmetric matrix such that for arbitrary vector $v\in R^{3}$ and vector cross product ×,

$$\hat{n}\left(\nu ,\lambda \right)v=n\left(\nu ,\lambda \right)\times v$$

and

$$n\left(\nu ,\lambda \right)=\left(\begin{array}{c} \sin\nu \cos\lambda \\ \sin\nu \sin\lambda \\ \cos\nu \end{array}\right).$$

There are several different ways to choose the ranges of these coordinates to fully parameterize SO(3). One way is to view these coordinates as a solid ball of radius π in which θ∈[0,π] serves as the radius and ν∈[0,π] and λ∈[0,2π), are the usual spherical angles. Another way is to let θ∈[0,2π) and cut the range of one of the other variables in half. For example, ν∈[0,π/2] and λ∈[0,2π) would restrict n to the upper hemisphere and ν∈[0,π] and λ∈[0,π) would be like the western hemisphere (if the initial datum is chosen appropriately). In these hemispherical boundary models, the great circle that bounds the hemisphere will be half open and half closed so as not to redundantly parameterize.

There are benefits to each of these parameterizations. For example, allowing the [0,2π) range for θ reflects that for fixed n rotations around a fixed axis bring back to the same location. That is the ‘little group’ of rotations around n isomorphic to SO(2) is the ‘maximal torus’ in SO(3). Likewise parameterizing the whole sphere has value. For this reason, the best of both worlds can be achieved by double covering rotations by allowing both ranges to be expanded. Moreover, each range [0,2π) can be replaced with [−π,π). Then when performing integration all that needs to be done is to divide by 2 afterwards.

Using these parameters, the integration measure dR such that the volume of SO(3) is normalized to 1 is

(74) $$dR\;=\;\frac{1}{4\pi^{2}}\sin^{2}\left(\theta /2\right)\sin\nu \;d\theta d\lambda d\nu \;.$$

When computing integrals,

$$\begin{array}{ccc} \int_{SO(3)}f\left(R\right)\;dR\; & = & \;\frac{1}{2\pi^{2}}\int_{\nu =0}^{\pi }\int_{\lambda =0}^{2\pi }\int_{\theta =0}^{\pi }f\left(R\left(\theta ,\nu ,\lambda \right)\right)\sin^{2}\left(\theta /2\right)\sin\nu \;d\theta d\lambda d\nu \\ \; & = & \;\frac{1}{2\pi^{2}}\int_{\nu =0}^{\pi /2}\int_{\lambda =0}^{2\pi }\int_{\theta =-\pi }^{\pi }f\left(R\left(\theta ,\nu ,\lambda \right)\right)\sin^{2}\left(\theta /2\right)\sin\nu \;d\theta d\lambda d\nu \\ \; & = & \;\frac{1}{2\pi^{2}}\int_{\nu =0}^{\pi }\int_{\lambda =0}^{\pi }\int_{\theta =-\pi }^{\pi }f\left(R\left(\theta ,\nu ,\lambda \right)\right)\sin^{2}\left(\theta /2\right)\sin\nu \;d\theta d\lambda d\nu \end{array}$$

Doubling the range gives

(75) $$\int_{SO(3)}f\left(R\right)\;dR\;=\;\frac{1}{4\pi^{2}}\int_{\nu =0}^{\pi }\int_{\lambda =0}^{2\pi }\int_{\theta =-\pi }^{\pi }f\left(R\left(\theta ,\nu ,\lambda \right)\right)\sin^{2}\left(\theta /2\right)\sin\nu \;d\theta d\lambda d\nu$$

and when f(R(θ,ν,λ))=f(θ)=f(−θ)

(76) $$\begin{array}{ccc} \int_{SO(3)}f\left(R\right)\;dR\; & = & \;\frac{2}{\pi }\int_{\theta =0}^{\pi }f\left(\theta \right)\sin^{2}\left(\theta /2\right)\;d\theta \\ \; & = & \;\frac{1}{\pi }\int_{\theta =-\pi }^{\pi }f\left(\theta \right)\sin^{2}\left(\theta /2\right)\;d\theta \;. \end{array}$$

All normalizations are such that:

$$\int_{SO(3)}1\;dR\;=\;1\;.$$

The Laplacian operator for SO(3) in this axis-angle parameterization is [3,97,98]

(77) $$\nabla^{2}=\frac{\partial^{2}}{\partial \theta^{2}}+\cot\theta /2\frac{\partial }{\partial \theta }+\frac{1}{4\sin^{2}\theta /2}\left(\frac{\partial^{2}}{\partial \nu^{2}}+\cot\nu \frac{\partial }{\partial \nu }+\frac{1}{\sin^{2}\nu }\frac{\partial^{2}}{\partial \lambda^{2}}\right)$$

It can be shown that the isotropic solution does not depend on ν or λ, and so all that needs to be solved is [99,100]:

(78) $$\frac{\partial f}{\partial t}=K\left(\frac{\partial^{2}f}{\partial \theta^{2}}+\cot\theta /2\frac{\partial f}{\partial \theta }\right)$$

subject to initial condition f(R,0)=δ(R).

A basis for all functions on SO(3) that depend only on θ are the functions $\left\{\chi_{l}\left(\theta \right)\;|\;l\in Z_{\geq 0}\right\}$ where

$$\chi_{l}\left(\theta \right)\;=\;\frac{\sin(l+\frac{1}{2})\theta }{\sin\frac{\theta }{2}}\;.$$

These are eigenfunctions of the Laplacian:

$$\nabla^{2}\chi \;=\;-l\left(l+1\right)\chi \;.$$

Consequently, the Fourier series solution of the isotropic heat equation on SO(3) is known to be:

(79) $$\;f\left(R\left(\theta ,\nu ,\lambda \right),t\right)\;=\;\sum_{l=0}^{\infty }\left(2l+1\right)\chi_{l}\left(\theta \right)\;e^{-l(l+1)Kt}\;=\;{\left(\sin\frac{\theta }{2}\right)}^{-1}\sum_{l=0}^{\infty }\left(2l+1\right)\;\sin\left((l+1/2)\theta \right)\;e^{-l(l+1)Kt}\;.\;$$

Note that:

$$\lim_{t\to \infty }f\left(R\left(\theta ,\nu ,\lambda \right),t\right)\;=\;1$$

and

$$\int_{SO(3)}f\left(R,t\right)\;dR\;=\;1\;.$$

When t=0, the above becomes the Fourier series for the Dirac delta function

f(R,0)=δ(R).

As with the case of the heat equation on the circle, an alternative solution exists, analogous to a folded Gaussian. Denote this solution as ρ(θ,t), and

(80) $$f\left(R\left(\theta ,\nu ,\lambda \right),t\right)=e^{Kt/4}{\left(\sin\frac{\theta }{2}\right)}^{-1}\sum_{k\in Z}\rho \left(\theta +2\pi k,t\right)\;.$$

This can be derived in two steps. First, let

$$f\left(R\left(\theta ,\nu ,\lambda \right),t\right)\;=\;{\left(\sin\frac{\theta }{2}\right)}^{-1}h\left(\theta ,t\right)\;.$$

Substituting in to (78) and simplifying then gives

(81) $$\frac{\partial h}{\partial t}=K\left(\frac{\partial^{2}h}{\partial \theta^{2}}+\frac{1}{4}h\right)\;.$$

Then substituting $h\left(\theta ,t\right)=e^{at}q\left(\theta ,t\right)$ and simplifying gives that when a=K/4

(82) $$\frac{\partial q}{\partial t}=K\frac{\partial^{2}q}{\partial \theta^{2}}\;.$$

The fundamental solution of this heat Equation (the 1D heat kernel) is

$$q\left(\theta ,t\right)\;=\;\frac{1}{2\sqrt{\pi Kt}}e^{-\theta^{2}/4Kt}\;.$$

However, this solution is not a satisfactory choice for ρ(θ,t) for several reasons. First, SO(3) is a 3D space, and so the normalization is not correct, as the normalization factor should be proportional to $1/t^{3/2}$. However, to arbitrarily change the temporal dependence will cause the result to no longer be a solution of (82). Secondly, whereas division by sin(θ/2) is fine in the definition of $\chi_{l}\left(\theta \right)$ because zeros are balanced by zeros in the numerator, that is not the case here.

There is a way to solve both problems. That is by realizing that if q(θ,t) solves (82), then so too does C∂q/∂θ for arbitrary constant *C*. Consequently, we take as a candidate solution [99,100]:

(83) $$\rho \left(\theta ,t\right)=C\frac{1}{{\left(\pi Kt\right)}^{3/2}}\theta e^{-\theta^{2}/4Kt}\;.$$

The choice of normalizing factor *C* is made such that (80) is a pdf. This is a constant, hence independent of *t*. Choosing a relatively small value of *t*, the summation in (80) reduces to a single term and (76) becomes

$$\frac{1}{\pi }C\frac{e^{Kt/4}}{{\left(\pi Kt\right)}^{3/2}}\int_{\theta =-\pi }^{\pi }\theta e^{-\theta^{2}/4Kt}\sin\left(\theta /2\right)\;d\theta \;=\;1\;.$$

Moreover, for small *t* the integral over [−π,π] can be replaced with an integral over the whole real line. Consequently, since

$$\int_{-\infty }^{\infty }\theta e^{-\theta^{2}/4Kt}\sin\left(\theta /2\right)\;d\theta \;=\;2\sqrt{\pi }{\left(Kt\right)}^{3/2}e^{-Kt/4}$$

then

$$C=\pi^{2}/2$$

and

(84) $$\;f\left(R\left(\theta ,\nu ,\lambda \right),t\right)=\frac{\sqrt{\pi }}{2}\frac{e^{Kt/4}}{{\left(Kt\right)}^{3/2}}{\left(\sin\frac{\theta }{2}\right)}^{-1}\sum_{k\in Z}\left(\theta +2\pi k\right)\;e^{-{\left(\theta +2\pi k\right)}^{2}/4Kt}\;.\;$$

Since f(R,t)>0 and integrates to 1 it is a valid pdf. What needs to be tested is

$$\lim_{t\to \infty }f\left(R,t\right)\;=\;1$$

and

$$\lim_{t\to 0}f\left(R,t\right)\;=\;\delta \left(R\right)\;.$$

If these conditions hold, then the solution is valid.

This provides a way to bound entropy in a way similar to the case of the circle. The next section makes more general exact statements for all values of time.

### 5.4. Rate of Entropy Production under Diffusion on Unimodular Lie Groups

The entropy of a pdf on a Lie group is defined in (5). If f(g,t) is a pdf that satisfies a diffusion equation, then some interesting properties of $S_{f}\left(t\right)$ that are independent of initial conditions result. For example, if ${\dot{S}}_{f}=dS_{f}/dt$, then differentiating under the integral gives

$$\begin{array}{ccc} {\dot{S}}_{f} & = & -\int_{G}\left\{\frac{\partial f}{\partial t}\log f+\frac{\partial f}{\partial t}\right\}\;dg. \end{array}$$

Moreover, since *f* is a pdf,

$$\int_{G}\frac{\partial f}{\partial t}\;dg=\frac{d}{dt}\int_{G}f\left(g,t\right)\;dg=0\;.$$

and so the second term in braces in the expression for ${\dot{S}}_{f}$ integrates to zero.

Substituting the diffusion equation

(85) $$\frac{\partial f}{\partial t}=\frac{1}{2}\sum_{i,j=1}^{n}D_{ij}{\tilde{E}}_{i}{\tilde{E}}_{j}f-\sum_{k=1}^{n}a_{k}{\tilde{E}}_{k}f$$

into the expression for ${\dot{S}}_{f}$ gives [5,6,31]

$$\begin{array}{ccc} \dot{S} & = & -\int_{G}\left\{\frac{1}{2}\sum_{i,j=1}^{n}D_{ij}{\tilde{E}}_{i}{\tilde{E}}_{j}f-\sum_{k=1}^{n}a_{k}{\tilde{E}}_{k}f\right\}\log f\;dg\\ & = & -\frac{1}{2}\sum_{i,j=1}^{n}D_{ij}\int_{G}\left({\tilde{E}}_{i}{\tilde{E}}_{j}f\right)\log f\;dg+\sum_{k=1}^{n}a_{k}\int_{G}\left({\tilde{E}}_{k}f\right)\;\log f\;dg\\ & = & \frac{1}{2}\sum_{i,j=1}^{n}D_{ij}\int_{G}\left({\tilde{E}}_{j}f\right)\left({\tilde{E}}_{i}\log f\right)\;dg-\sum_{k=1}^{n}a_{k}\int_{G}f\;\left({\tilde{E}}_{k}\log f\right)\;dg\\ & = & \frac{1}{2}\sum_{i,j=1}^{n}D_{ij}\int_{G}\frac{1}{f}\left({\tilde{E}}_{j}f\right)\left({\tilde{E}}_{i}f\right)\;dg-\sum_{k=1}^{n}a_{k}\int_{G}{\tilde{E}}_{k}f\;dg\\ & = & \frac{1}{2}\sum_{i,j=1}^{n}D_{ij}\int_{G}\frac{1}{f}\left({\tilde{E}}_{j}f\right)\left({\tilde{E}}_{i}f\right)\;dg\\ & = & \frac{1}{2}\mathrm{tr}\left[DF\right] \end{array}$$

where $F=[F_{ij}]$ is the Lie-group version of the Fisher information matrix with entries

$$F_{ij}\;≐\;\int_{G}\frac{1}{f}\left({\tilde{E}}_{j}f\right)\left({\tilde{E}}_{i}f\right)\;dg\;.$$

Consequently,

$$\frac{1}{2}\lambda_{min}\left(D\right)\mathrm{tr}\left[F\right]\;\leq \;\dot{S}\;\leq \;\frac{1}{2}\lambda_{max}\left(D\right)\mathrm{tr}\left[F\right]\;.$$

The above result is an extension of one presented in [5,6,31].

### 5.5. The Generalized de Briujn Identity

Here a theorem derived in [5,6,31] is restated.

**Theorem** **3.** *Let the solution of the diffusion Equation (85) with constant $a={\left[a_{1},...,a_{n}\right]}^{T}$ subject to the initial condition f(g,0;D,a)=δ(g) be denoted as $f_{D,a,t}\left(g\right)=f\left(g,t;D,a\right)$. Let α(g) be another differentiable pdf on the group. Then*

(86) $$\frac{d}{dt}S\left(\alpha *f_{D,a,t}\right)=\frac{1}{2}\mathrm{tr}\left[DF\left(\alpha *f_{D,a,t}\right)\right].$$

**Proof.**  The solution of the diffusion equation

(87) $$\frac{\partial \rho }{\partial t}=\frac{1}{2}\sum_{i,j=1}^{n}D_{ij}{\tilde{E}}_{i}{\tilde{E}}_{j}\rho -\sum_{k=1}^{n}a_{k}{\tilde{E}}_{k}\rho$$

subject to the initial conditions ρ(g,0)=α(g) is $\rho \left(g,t\right)=(\alpha *f_{D,a,t})\left(g\right)$. Then computing the derivative of S(ρ(g,t)) with respect to time yields

(88) $$\frac{d}{dt}S\left(\rho \right)=-\frac{d}{dt}\int_{G}\rho \left(g,t\right)\log\rho \left(g,t\right)\;dg=-\int_{G}\left\{\frac{\partial \rho }{\partial t}\log\rho +\frac{\partial \rho }{\partial t}\right\}\;dg.$$

By substituting in (87), the partial with respect to time can be replaced with Lie derivatives. However,

$$\int_{G}{\tilde{E}}_{k}\rho \;dg=\int_{G}{\tilde{E}}_{i}{\tilde{E}}_{j}\rho \;dg=0\;.$$

Consequently, the second term on the right side of (88) completely disappears. Using the integration-by-parts formula (There are no surface terms. As with the circle and real line, each coordinate in the integral either wraps around or goes to infinity.)

$$\int_{G}f_{1}\;{\tilde{E}}_{k}f_{2}\;dg=-\int_{G}f_{2}\;{\tilde{E}}_{k}f_{1}\;dg,$$

with $f_{1}=\log\rho$ and $f_{2}=\rho$ then gives

$$\begin{array}{ccc} \frac{d}{dt}S\left(\alpha *f_{D,a,t}\right) & = & \frac{1}{2}\sum_{i,j=1}^{n}D_{ij}\int_{G}\frac{1}{\alpha *f_{D,a,t}}{\tilde{E}}_{j}\left(\alpha *f_{D,a,t}\right){\tilde{E}}_{i}\left(\alpha *f_{D,a,t}\right)\;dg\\ & = & \frac{1}{2}\sum_{i,j=1}^{n}D_{ij}F_{ij}\left(\alpha *f_{D,a,t}\right)\;=\;\frac{1}{2}\mathrm{tr}\left[D\;F(\alpha *f_{D,a,t})\right]. \end{array}$$

This means that:

$$S\left(\alpha *f_{D,a,t_{2}}\right)-S\left(\alpha *f_{D,a,t_{1}}\right)=\frac{1}{2}\int_{t_{1}}^{t_{2}}\mathrm{tr}\left[DF(\alpha *f_{D,a,t})\right]dt.$$

□

Whereas some inequalities of Information Theory generalize to the Lie group setting, as demonstrated above, others do not. For example, under convolution on a Lie group (24) and (25) do not hold in general. As for the CRB, there are versions for Lie groups applicable to small values of Dt or with different concepts of covariance, but not in a way that is directly applicable to the scenarios formulated here.

## 6. Conclusions

Stochastic mechanical systems can describe individual representatives of a statistical mechanical ensemble (as in a rotor in rotational Brownian motion), or can be a stand-alone system subjected to noise, such as a kinematic cart robot. When these systems have mass, Itô and Stratonovich SDEs lead to the same Fokker–Planck equation. Even in the case of inertia-free kinematic systems evolving on Lie groups, these can be the same. From the Fokker–Planck equation, the rate of entropy can be computed. For diffusion processes (with or without drift), the rate of entropy increase is related simply to the diffusion matrix and the Fisher information matrix. This result holds both in Euclidean spaces and on unimodular Lie groups (including cotangent bundle groups), which are a common configuration space for mechanical systems. As systems approach equilibrium, the entropy rate approaches zero. Two different ways to approach equilibrium are discussed: (1) when there is a restoring potential; (2) when the configuration space is bounded. By using the monotonicity and convexity properties of the logarithm function together with inequalities from information theory, computable bounds on entropy and entropy rate are established.

## Data Availability

Not applicable.
